# Supplementary material for: Genetic Characterization and Pathogenesis of Avian Influenza Virus H3N8 Isolated from Chinese pond heron in China in 2021
Source: Viruses. 2023 Jan 28;15(2):383. doi: 10.3390/v15020383 (PMC9966531; doi:10.3390/v15020383)
Supplement: Supplementary file 1 [file viruses-15-00383-s001.zip › viruses-2103027-supplementary.pdf]

Supplementary data

Genetic Characterization and Pathogenesis of Avian Influenza Virus H3N8 Isolated from Chinese pond heron in China in 2021

Heng Zhang [1,†], Shuyi Han [2,3,†], Bo Wang [2,3], Yanan Xing [2,3], Guohui Yuan [2], Ye Wang [4], Zhilei Zhao [2], Gaojian Li [2,3], Qiaoqiao Li [1], Jinchao Pan [1], Wenchao Li [1,*] and Hongxuan He [2,*]

Table S1. Primer sequences used in this study.

Primer name	Sequence (5'→3')	Application	Expected size (bp)	Reference
Ba-HA-1	F: TATTGGTCTCAGGGAGCGAAAGCAGGGG	Hemagglutinin (HA) gene specific primers	1778	[1]
Ba-NS-89OR	R: ATATGGTCTCGTATTAGTAGAAACAAGGG TGTTTT			
Ba-NA-1	F: TATTGGTCTCAGGGAGCGAAAGCAGGAGT	Neuraminidase (NA) gene specific primers	1413	[1]
Ba-NA-1413R	R: ATATGGTCTCGTATTAGTAGAAACAAGGA GTTTTTT			
MBTuni-12	ACGCGTGATCAGCAAAAGCAGG	A universal primer for whole genome amplification of influenza A virus	800-2300	[2]
MBTuni-13	ACGCGTGATCAGTAGAAACAAGG			
M13	F: TGTAAAACGACGGCCAGT	PCR primer for colony identification	-	-
	R: CAGGAAACAGCTATGACC			
GAPDH	F: ACCACCATGGAGAAGGCTGG	Standardized internal parameters	-	-
	R: CTCAGTGTAGCCCAGGATGC			
M229L	F: GACCA ATCCTGTCACCTCTGA	Universal primers for the conserved region of influenza A virus M gene	229	[3]
M229U	R: GTATATGAGGCCCATRCAACT			

Table S2. Whole genome sequence of JX 5-1.

Gene	Length	sequence
PB2	2287	GCATTAATTCATATGGAGAGATAAGAGAATTAAGAGATTTGATGTCACAGTCTCGCACTCGCGAGATACTGACAA<br>AAACCACTGTGGACCATATGGCCATAATTAAGAAATACACGTCAGGAAGACAGGAGAAGAACCCTGCCCTTAGG<br>ATGAAATGGATGATGGCAATGAAATATCCGATTACAGCAGACAAAAGAATAATGGAAATGATCCCTGAAAGAA<br>ATGAACAAGGCCAGACTCTCTGGAGCAAAACAAATGATGCTGGATCAGACAGAGTGATGGTGTCACCCCTGGCT<br>GTGACATGGTGGAATAGAAATGGGCCGACGACGAGTACGGTTCATTACCCAAAGGTCTACAAAACCTACTTTGA<br>AAAAGTCGAAAGGTTGAAGCATGGAACCTTCGGTCCTGTTCACTTTCGGAACCAGGTTAAAATACGCCGCAGGGT<br>TGACATAAACCCGGGCCATGCAGATCTCAGTGCCAAAGAAGCACAAGATGTCATCATGGAGGTTGTTTTCCCAAA<br>TGAAGTTGGAGCCAGGATATTGACATCAGAATCACAATTAACAATAACAAAAGAAAAGAAAGAGGAGCTTCAG<br>GACTGTAAGATCGCCCCTTTAATGGTGGCATACATGTTGGAGAGAGAACTGGTTCGCAAAACCAGATTCCTACCA<br>GTAGCTGGCGGACAAGCAGCGTGTACATCGAGGTATTGCATTTGACTCAAGGGACCTGCTGGGAACAGATGTAC<br>ACACCGGGAGGGGAGGTGAGAAATGATGATGTCGATCAGAGTTTGATCATTGCTGCTAGAAATATTGTTAGGAGA<br>GCAACAGTATCAGCAGACCCGTTGGCTTCGCTCCTGGAAATGTGCCATAGTACACAAATTGGCGGGATAAGAATG<br>GTGGACATTCTTAGACAGAACCCGACAGAAGAGCAAGCTGTGGATATATGCAAAGCAGCAATGGGTTTGAGAAT<br>CAGTTCATCCTTCAGCTTTGGAGGTTTCACTTTTAAAAGGACAAGTGGGTCATCCATCAAAAGAGAAGAGGAAGT<br>GCTCACAGGCAACCTTCAAACACTGAAAATAAGAGTACATGAAGGATATGAAGAATTCACAATGGTTGGACGAA<br>GAGCTACAGCCATTCTAAGGAAAGCAACCAGGAGGCTGATCCAATTAATAGTGAGTGGGAGAGACGAACAATCA<br>ATCGCTGAAGCGATTATAGTGGCAATGGTTTTCTCGCAAGAGGATTGCATGATAAAAGCAGTACGAGGTGATTTG<br>AACTTTGTTAACAGAGCAAATCAGCGGCTGAATCCTATGCATCAACTTCTGAGGCATTTCCAAAAGGATGCAAAG<br>GTGTTGTTTCAGAATTGGGGAATTGAATCCATTGACAATGTCATGGGAATGATAGGAATACTGCCTGACATGACC<br>CCCAGCACAGAGATGTCACTGAGAGGAGTGAGAGTCAGTAAAATGGGAGTGGATGAATACTCCAGTACTGAGAG<br>AGTGGTCGTGAGCATTGATCGTTTCTTGAGAGTCCGAGACCAGAGGGgAAACGTGCTCtTgTCTCCTGAAGAGGTT<br>AGTGAAACACAGGGAACAGAGAAATTGACGATAACATATTCATCGTCCATGATGTGGGAAATCAATGGTCCGGA<br>GTCAGTGCTGATCAACACATACCAATGGATCATTAGAAATTGGGAAACTGTGAAGATTCAGTGGTCCCAAGACCC<br>TACAATGCTGTACAATAAGATGGAGTTTGAGCCTTTCCAATCCTTGGTGCCCAAGGCTGCCAAAGGCCAGTATAG<br>TGGATTTGTGAGGACGTTATTCCAGCAGATGCGTGATGTGCTGGGGACATTTGACACTGTCCAAATAATAAAGCTC<br>TTACCATTTGCAGCAGCCCCACCGGAACAGAGTAGGATGCAGTTCTCTTCTCTGACTGTGAATGTAAGAGGCTCA<br>GGCATGAGAATACTTGTGAGAGGCAACTCCCCTGTGTTCAACTTTAACAAAACAACCAAAAGACTCACAGTTCTT<br>GGAAAGGATACAGGTGCATTGACAGAAGATCCAGATGAGGGAACAGCAGGAGTGGAATCTGCAGTATTAAGAG<br>GATTTCTAATTCTGGGCAAAGAAGACAAAAGATATGGACCAGCATTGAGCATTAACGAGTTGAGCAATCTTGCGA<br>AAGGGGAGAAGGCTAATGTGTTGATAGGGCAAGGAGACGTGGTGTTGGTAATGAAACGGAAACGGGACTCTAGC<br>ATACTTACTGACAGCCAGACAGCGACCAAAAGAATTCGGATGGCCATCAA
PB1	2309	CCCGTTTGATGGATGTTATCCGACTTTACTTTTCTTAAAAGTGCCGGCGCAAAATGCCATAAGTACCACATTTCCTT<br>ATACTGGAGATCCTCCATACAGCCATGGAACAGGAACAGGATACACCATGGACACAGTCAACAGAACACATCAA<br>TACTCAGAGAAGGGGAAATGGACAACAAACACAGAGACCGGAGCACCCCAACTTAATCCAATTGATGGACCAC<br>TACCTGAGGACAACGAACCAAGCGGATATGCACAAACAGATTGCGTATTGGAAGCAATGGCTTTCCTTGAAGAG<br>TCCCACCCAGGGATCTTTGAAAACTCTTGTCTTGAAACGATGGAAGTTGTTCAGCAAACAAGAGTGGACAAACTA<br>ACTCAAGGTCGCCAGACTTATGACTGGACGCTGAATAGAAACCAACCAGCTGCAACTGCCCTGGCCAACACTAT<br>AGAAGTCTTCAGATCGAATGGTCTGACAGCCAATGAATCGGGGAGACTAATAGATTTCCTCAAGGATGTGATGGA

		CTCAATGGACAAAGAGGAAATGGAAATAACAACACATTTCCAGAGAAAGAGAAGAGTAAGGACAACATGACC<br>AAGAAAATGGTCACACAAAGAACAATAGGAAAGAAGAAACAGAGGCTGAATAAGAAGAGCTACTTAATAAGA<br>GCTCTGACACTAAACACAATGACAAAAGATGCAGAAAGAGGCAAATTGAAGAGGCGGGCGATTGCAACACCAG<br>GGATGCAGATTAGAGGATTTGTCTACTTTGTTGAAACACTGGCGAGGAGTATCTGTGAGAAACTTGAGCAATCTG<br>GACTCCCCGTTGGAGGGAATGAGAAAAAGGCTAAATTGGCAAATGTCGTGAGAAAAATGATGACCAACTCGCAA<br>GATACAGAGCTCTCTTTCACAATTACTGGAGATAACACCAAATGGAATGAGAATCAGAATCCTCGGATGTTTCTG<br>GCAATGATAACATACATCACAAGAAACCAACCTGAATGGTTTAGAAATGTCTTGAGCATTGCCCCTATAATGTTC<br>TCGAATAAAATGGCGAGATTGGGAAAAGGATACATGTTTGAAAGTAAGAGCATGAAGCTACGGACACAAATACC<br>TGCAGAAATGCTTGCAAACATTGACTTGAAATATTTCAATGAATCAACAAGAAAGAAAATCGAGAAAATAAGAC<br>CTCTACTAATAGATGGCACAGCCTCATTGAGCCCTGGAATGATGATGGGCATGTTCAATATGCTGAGTACAGTATT<br>AGGAGTCTCGATCCTGAATCtTGGACAAAAGAgGTACACCAAAACCACATACTGGTGGGATGGACTTCAATCCTCT<br>GATGATTTCGCCCTCATAGTGAATGCACCGAATCATGAGGGAATACAAGCAGGAGTGGATAGGTTCTATAGGACC<br>TGCAAGCTGGTTGGAATCAACATGAGCAAAAAGAAGTCTTACATAAACCGGACAGGAACATTTGAATTCACAAG<br>CTTTTTCTATCGCTATGGGTTTGTGGCTAATTTCAGTATGGAGCTGCCCAGCTTTGGAGTGTCTGGAATCAATGAAT<br>CAGCTGACATGAGCATTGGAGTTACAGTGATAAAGAATAACATGATAAACAATGACCTTGGACCAGCAACAGCT<br>CAGATGGCTCTTCAGCTATTCATCAAGGACTACAGATACACGTACCGATGCCACAGGGGTGACACACAAATTCAA<br>ACGAGGAGATCATTTGAGCTGAAGAAGCTATGGGAGCAGACCCGTTCAAAGGCAGGACTGTTGGTGTCGGATGG<br>AGGACCAAACCTATACAACATTCGGAATCTCCACATCCCAGAGGTCTGCTTGAAGTGGGAGCTGATGGACGAAG<br>ATTACCAGGGCAGGTTGTGTAATCCCCTGAATCCATTTGTCAGTCATAAGGAAATTGAGTCCGTAAACAATGCTGT<br>GGTGATGCCAGCCCATGGCCCAGCCAAGAGCATGGAATATGATGCTGTTGCGACTACACACTCATGGATTCCTAA<br>GAGGAACCGTTCCATTCTCAATACCAGCCAAAGGGGAATTCTTGAGGATGAGCAGATGTACCAGAAGTGCTGTA<br>GTCTATTCGAGAAATTCTTCCCCAGTAGTTCATACAGGAGGCCAGTTGGAATTTCCAGCATGGTGGAGGCCATGGT<br>ATCTAGGGCCCGAATTGATGCACGCATTGATTTCGAATCTGGAAGGATTAAGAAAGAAGAGTTTGCTGAGATCAT<br>GAAGATCTGTTCCACCATTGAAGAGCTCAGACGGCAGAAATAGTGAATTTAGCTGTCATCATTAAAAAAAG
PA	2130	CAGAAATCAATGGAGACTTTGTGCGACAATGCTTCAATCCAATGATTGTCGAGCTTGCGGAAAAGGCAATGAAA<br>GAATATGGGGAAGATCCGAAAATCGAAACAAATAAATTTGCCGCAATATGCACTCACTTAGAAGTCTGTTTCATG<br>TATTCGGATTTCCACTTTATTGATGAACGAGGCGAATCAATAATTGTAGAATCTGGCGATCCGAATGCATTATTGA<br>AGCACCGATTCGAGATAATTGAAGGGAGAGACCGAACAATGGCCTGGACAGTGGTGAATAGTATCTGCAACACC<br>ACAGGAGTCGAAAAGCCCAAATTCCTCCCTGATTTGTATGACTACAAAGAGAACCGATTCATTGAAATTGGAGTT<br>ACGCGAAGGGAAGTTCACATATACTATCTAGAAAAAGCCAACAAGATAAAGTCGGAGAAGACACACATTCACA<br>TATTCTCATTCACTGGAGAGGAAATGGCCACCAAGGCGGACTACAGCCTTGATGAAGAAAGCAGAGCAAGAATA<br>AAAACCAGGCTGTTCACTATAAGACAAGAAATGGCCAGTAGGGGTCTATGGGATTCCTTTCGTCAGTCCGAGAGA<br>GGCGAAGAGACAATTGAAGAAAGATTTGAAATCACAGGAACCATGCGCAGGCTTGCCGACCAAA<br>GTCTCCCACCGAACTTCTCCAGCCTTGAAAACTTTAGAGCCTATGTGGATGGATTCGAACCGAACGGCTGCATTGA<br>GGGCAAGCTTTCTCAGATGTCAAAAGAAGTGAATGCCAGAATTGAGCCGTTTCTGAAGACAACACCACGCCCTCT<br>TAGATTACCTGATGGGCCTCCTTGTTCTCAGCGATCGAAGTTCTTGCTGATGGATGCCCTCAAATTGAGCATCGAA<br>GACCCGAGTCATGAGGGGGAGGGTATACCATTGTATGATGCAATCAAATGTATGAAGACATTTTTTGGCTGGAAA<br>GAACCCACCATCATAAAACCACATGAAAAAGGCATAAACCCTAATTACCTCCTGGCTTGGAAGCAGGTGCTGAC<br>AGAACTCCAAGATATTGAAAATGAGGAGAAAATCCCAAAAACAAAGAACATGAAGAAAACAAGCCAATTGAA<br>GTGGGCACTTGGTGAGAACATGGCACCAGAAAAAGTGGACTTTGAGGATTGTAAAGATGTTAGCGACTTAAAAC<br>AGTACGACAGTGACGAACCAGAGCCTAGATCACTAGCAAGCTGGATCCAGAGTGAATTCAACAAGGCATGCGAA<br>TTAACAGATTCaAGTTGGATTGAACTTGATGAAATAGGAGAAGACGTTGCTCCAATTGAGCATA

		TTGCGAGTATGAGGAGGAACTATTTCACAGCAGAAGTATCCCATTGCAGGGCTACTGAATACATAATGAAGGGA<br>GTATACATAAACACAGCCCTATTGAATGCATCCTGTGCAGCCATGGATGACTTCCAATTGATTCCAATGATAAGC<br>AAATGCAGAACCAAGGAAGGAAGACGGAAGACAAATCTGTATGGGTTCATTGTAAAAGGAAGATCCCATTTGAG<br>GAATGATACCGATGTGGTAAACTTTGTGAGCATGGAATTCTCTCTCACTGACCCGAGGCTGGAGCCACATAAATG<br>GGAAAAGTACTGTGTTCTTGAGATAGGGGACATGCTTCTACGGACTGCAATAGGCCAAGTGTCAAGGCCCATGTT<br>CCTGTATGTGAGAACCAATGGGACCTCCAAGATCAAAATGAAATGGGGCATGGAGATGAGGCGATGCCTTCTTC<br>AGTCCCTTCAACAAATTGAAAGCATGATTGAGGCCGAGTCTTCTGTTAAAGAGAAGGACATGACCAAGGAATTCT<br>TTGAAAACAAATCAGAAACATGGCCAATTGGTGAATCGCCCAAAGGGGTAGAAGAAGGCTCCATTGGGAAGGTG<br>TGCAGAACATTGTTAGCCAAGTCTGTGTTCAACAGCCTGTATGCATCTCCACAGCTCGAGGGGT<br>TTTCAGCTGAATCAAGAAAATTGCTTCTCATTGTTCAGGCACTTAGGGACAACCTGGAACCTGGAACCTTCGATCT<br>TGGGGGGCTATATGAAGCAATTGAGGAGTGCCTGATTAACGATCCCTGGGTTTTGCTTAATGCGTCTTGGTTCA
HA	1800	TAGCGGCCGCGAATTCGCCCTTCGCGTGATCAGCAAAAGCAGGGGATACTTTCATTAATCATGAAGACCATTATC<br>GCATTGAGCTACATTCTCTGCTTGGCTTTTGGACAGAACCTTTCAGGGAATGACAGCAGTACAGCAACACTATGCC<br>TGGGACATCATGCAGTGCCGAATGGAACAATAGTGAAAACAATCACCGATAACCAGATTGAGGTGACCAATGCT<br>ACTGAGCTGGTTCAGAGTTCCTCAACAGGGAAAATATGTAACAATCCCCACAGAATCCTTGATGGAAGGGACTGC<br>ACATTAATAGATGCCATGCTTGGAGATCCTCATTGCGATGTTTTTCAAGATGAGACCTGGGATCTCTTTATTGAGC<br>GAAGCAATGCTTTCAGCAATTGTTATCCCTATGATGTGCCGGATTATGCCTCCCTTCGATCTCTAGTTGCTTCATCA<br>GGCACGCTAGAATTCATTACCGAAGGTTTCACCTGGGCAGGAGTGAGCCAGAATGGAGGGAGCGGTGCCTGCAA<br>AAGAGGACCTGCCAACGGTTTCTTTAGTAGGTTAAACTGGTTGACCAAGTCAGGAAATTCATACCCACTATTAAA<br>CGTGACTATGCCAAACAATGACAATTTTGACAGGTTATACATCTGGGGTGTTCACCACCCAAGTACAAACCAAGA<br>ACAGACTAATCTATATGTTCAGGCCTCAGGAAGAGTCACAGTCTCCACCAGGAGAAGTCAACAAACCATAGTCC<br>CGAACATTGGATCTAGACCTTGGGTGAGGGGTCAATCTGGCAGGATAAGCATCTACTGGACAATAGTCAAACCTG<br>GAGACGTACTGGTAATCAACAGTAATGGAAATCTAATTGCGCCTCGTGGATACTTCAAGATCCGAACTGGGAAAA<br>GCTCAATAATGAGGTCAGATGCACCTATAGAAACCTGCATCTCAGAATGCATTACTCCGAATGGAAGCATCCCTA<br>ATGACAAGCCTTTTCAAAATGTAAACAGAATCACATACGGGGCATGCCCCAAATACGTGAAGCAGAACACCCTA<br>AAATTGGCCACAGGAATGAGGAATGTGCCTGAGAAGCAAACCAGAGGTCTATTCGGTGCAATAGCAGGATTCAT<br>AGAGAATGGATGGGAAGGGATGATAGATGGTTGGTATGGCTTCAGGCATCAAAATTCTGAAGGCACAGGACAAG<br>CAGCAGATCTTAAAAGCACTCAAGCGGCCATTGATCAAATCAATGGGAAATTGAACAGGGTGATTGAAAAGACA<br>AATGAAAAATTCCATCAAATCGAGAAGGAATTCTCCGAGGTTGAAGGAAGGATTCAAGATCTTGAGAAATACGT<br>TGAAGACACAAAAGTGGATCTCTGGTCCTATAATGCAGAGCTTCTTGTTGCTCTAGAGAACCAACATACAATTGA<br>TTTGACCGATTCTGAGATGAATAAATTGTTTGAAAAAACCAGAAGGCAACTGAGGGAGAATGCTGAAGACATGG<br>GCAATGGTTGCTTCAAAATATACCACAAATGTGACAATGCCTGCATAGAATCAATTAGGAATGGAACTTATGACC<br>ATGACATATACAGAAATGAGGCACTGAACAATCGGTTCCAGATCAGGGGTGTTGAACTAAAATCTGGATACAAA<br>GACTGGATCCTGTGGATTTCCTTTGCCATATCATGCTTTTTGCTTTGTGTTGTGTTGTTGGGGTTCATTATGTGGGCTT<br>GCCAGCGGGGCAACATTAGGTGCAACATTTGCATTTGAGTATACTAATAATTAAAAACACCCTTGTTTCTACTATC<br>C
NP	1565	AGCAAAAGCAGGGTAGATAATCACTCACTGAGTGACATCAACATCATGGCGTCTCAAGGCACCAAACGATCTTA<br>CGAACAGATGGAAACTGGTGGGGAGCGCCAGAATGCCACTGAGATCAGAGCGTCTGTTGGAAGGATGGTTGGAG<br>GAATTGGAAGATTTTACATACAGATGTGCACCGAACTCAAACTCAGCGACTATGAAGGAAGGCTGATCCAGAAC<br>AGCATAACAATAGAGAGAATGCTTCTCTCTGCATTTGATGAAAGGAGGAACAAATACTTGGAAGAACACCCCAG<br>TGCGGGGAAAGACCCAAAGAAAACTGGAGGTCCAATTTACCGAAGGAGAGATGGGAAATGGATGAGAGAACTG

		ATTCTGTATGATAAAGAAGAGATCAGAAGGATCTGGCGTCAAGCGAATAATGGGGAAGATGCAACTGCTGGTCT<br>CACTCACCTGATGATCTGGCACTCCAACCTAAATGATGCAACATACCAGAGAACAAGAGCTCTCGTGCGCACTGG<br>AATGGACCCCAGAATGTGCTCTCTGATGCAAGGATCAACTCTCCCAAGGAGATCTGGAGCTGCTGGAGCAGCAGT<br>AAAAGGAGTTGGGACAATGGTGATGGAACTAATTCGGATGATAAAGCGAGGAATCAATGATCGGAATTTCTGGA<br>GAGGCGAGAATGGACGAAGGACAAGAATTGCATATGAGAGAATGTGCAACATCCTCAAAGGGAAATTTCAAAC<br>AGCAGCACAAAGAGCAATGATGGACCAAGTACGAGAGAGCAGAAATCCTGGAAATGCTGAAATTGAAGATCTC<br>ATCTTTCTGGCACGTTCTGCACTCATCCTGAGAGGATCAGTGGCCCATAAGTCCTGTCTGCCTGCTTGTGTATATGG<br>ACTTGCTGTGGCCAGTGGATATGATTTTGAGAGAGAAGGATACTCTCTAGTTGGGATAGATCCCTTCCGCTTGCTT<br>CAAAACAGCCAGGTCTTCAGTCTCATTAGACCAAATGAGAATCCAGCACACAAGAGTCAACTGGTCTGGATGGC<br>ATGTCATTCTGCAGCATTTGAAGACCTGAGAGTTTCAAGTTTCATCAGAGGAACAAGAGTGATTCCAAGAGGACA<br>ACTGTCTACCAGAGGAGTTCAAGTTGCCTCAAATGAGAACATGGAAACGATGGATTCCAGCACTCTTGAATTGAG<br>AAGCAGATACTGGGCTATAAGAACTAGGAGTGGAGGAA<br>ACACCAACCAGCAGAGAGCATCTGCAGGACAAATCAGTGTGCAGCCTACTTTCTCAGTGCAGAGAAATCTCCCCT<br>TCGAAAGAGCGACCATCATGGCGGCATTCACAGGGAATACTGAAGGCAGAACATCCGACATGAGAACCGAAAT<br>CATAAGAATGATGGAAAGTGCCAGACCAGAGGATGTGTCTTTCCAGGGGCGGGGAGTCTTCGAGCTCTCGGACG<br>AAAAGGCAACGAACCCGATCGTGCCTTCCTTTGACATGAGTAACGAAGGATCTTATTTCTTCGGAGACAATGCAG<br>AGGAGTATGACAATTAAAGAAAAATACCCTTGTATCTACT
NA	1436	TTAATGATCAAATCAGATATAATAACCATTGGGTCTGTATCCCTAGGATTGGTAGTCCTTAATATTCTCCTACATAT<br>AGTTAGTATTACAGTAACAGTGTTGGTTCTCCCTGGAAACGGAAATAATGGAAGTTGCAATGAAACAATCATTAG<br>GGAATACAATGAAACAATAAGGGTTGAGAAGGTAACACAATGGCACAATACCAATGTCATTGAGTATATAGAGA<br>GACCGGAGAATGATCATTTCATGAACAATACAGAAGCATTGTGTGATGCTAAGGGCTTCGCACCCTTTTCCAAAG<br>ACAACGGAATAAGAATTGGATCGAGAGGGCATGTTTTTGTCATAAGGGAGCCATTTGTTTCTTGCTCGCCAACAG<br>AGTGCAGAACGTTCTTCCTCACTCAAGGTTCCTTACTCAATGACAAACATTCTAATGGAACAGTTAAAGACCGGA<br>GCCCCTATAGAACTCTAATGAGTGTAGAAATAGGGCAATCACCCAATGTGTACCAGGCAAGGTTTGAAGCAGTG<br>GCGTGGTCAGCTACTGCATGTCATGACGGGAAGAAATGGATGACAATTGGAGTAACGGGCCCTGATGCCAAAGC<br>AGTGGCAGTGGTGCATTATGGGGGAATTCCTACTGATGTAATCAATTCCTGGGCAGGAGAT<br>ATTCTAAGAACTCAGGAATCATCATGCACTTGCATTCAAGGTGAATGTTTTTGGGTAATGACAGATGGACCAGCA<br>AATAGACAAGCGCAATACAGGGCGTTCAAAGCCAAGCAGGGGAAAATAGTCGGGCAAGCTGAAATCAGTTTCA<br>ATGGAGGCCATATAGAGGAATGCTCATGCTACCCCAATGAAGGTAAAGTGGAATGTGTTTGTAGGGACAATTGG<br>ACCGGAACCAATAGGCCAGTGTTGGTGATTTCTCCAGATTTGTCCTATAGAGTCGGGTACTTGTGTGCAGGTCTCC<br>CCAGTGACACCCCAAGAGGAGAAGATAGTCAGTTCACGGGATCATGCACTAGCCCAATGGGAAACCAGGGGTAC<br>GGAGTTAAGGGATTTGGATTCAGGCAGGGCAATGATGTATGGATGGGAAGGACCATTAGCAGAACATCAAGATC<br>GGGATTTGAGATCCTGAAAGTCAGAAATGGCTGGGTACAAAACAGTAAAGAGCAGATCAAAAGGCAAGTTGTGG<br>TCGATAATTTAAATTGGTCAGGATACAGTGGTTCTTTCACACTACCAGTGGAGTTAACAAAAAGGAATTGTCTGGT<br>TCCATGTTTTTGGGTTGAGATGATAAGGGGGAAGCCGGAAGAAAAGACGATATGGACCTCAAGTAGCTCCATTGT<br>GATGTGTGGAGTAGACCATGAGATTGCCGACTGGTCGTGGCACGATGGAGCTATTCTTCCTTTTGACATCGATAAG<br>ATGTAATTTACGAAAAAACTCCTTGTTTC
M	1570	AGCAAAAGCAGGTAGATGTTTAAAGATGAGTCTTCTAACCGAGGTCGAAACGTACGTTCTCTCTATCGTTCCGTCA<br>GGCCCCCTCAAAGCCGAGATCGCGCAGAGACTTGAAGATGTTTTTGCAGGGAAGAACACTGATCTTGAGGCTCTC<br>ATGGAATGGCTAAAGACAAGACCAATCTTGTCACCTCTGACCAAGGGGATTTTAGGATTTGTGTTCACGCTCACC<br>GTGCCCAGTGAGCGAGGACTGCAGCGTAGACGCTTTGTTCAGAATGCCCTGAATGGGAATGGAGACCCAAACAA

		CATGGATAGGGCAGTCAAACTGTACAGGAAGCTGAAGAGGGAAATAACATTCCATGGGGCCAAAGAGGTTGCA<br>CTTAGTTACTCAACCGGTGCACTTGCCAGTTGCATGGGTCTCATATACAACAGGATGGGAACAGTGACCACAGAA<br>GTGGCTTTTGGTCTGGTGTGCGCCACTTGTGAGCAGATTGCTGATTCACAGCATAGGTCTCACAGACAGATGGTGA<br>CTACCACCAACCCACTAATCAGGCATGAAAACAGAATGGTGCTGGCAAGCACTACAGCTAAAGCTATGGAGCAG<br>ATGGCTGGATCAAGTGAGCAAGCAGCAGAAGCCATGGAAGTTGCTAGTCAGGCTAGGCAGATGGTGCAGGCGAT<br>GAGAACAATTGGGACTCATCCTAGCTCCAGTGCCGGTCTGAAAGATGATCTTCTTGACAATTTGCAGGCCTACCA<br>GAAACGGATGGGAGTGCAAATGCAACGATTCAAGTGATCCTCTCGTTATTGCAGCAAGTATCATTGGGATCTTGC<br>ACTTGATATTGTGGATTCTTGATCGTCTTTTTTTCAAATGCACTTATCGTCGCCTTAAATACGGTTTGAAAAGAGGG<br>CCTTCTACGGAAGGAGTGCCTGAGTCTATGAGGGAAGAATATCGGCAGGAGCAACAGGATGCTGTGGACGTTGA<br>CGATGGTCATTTTGTCAACATAGAGCTGGAGTAAAAAACTACCTTGTTTCTACTGATCACGCGTAAGGGCGAATTC<br>GTTTAAACCTGCAGGACTAGTACCTTTAGTGAGGGTTAATTCTGAGCTTGGCGTAATCATGGTCATAGCTGTTTCCT<br>GTGTGAAATTGTTATCCGCTCACAATTCCACACAACATACGAGCCGGAAGCATAAAGTGTAAAGCCTGGGGTGCC<br>TAATGAGTGAGCTAACTCACATTAATTGCGTTGCGCTCACTGCCCGCTTTCCAGTCGGGAAACCTGTCGTGCCAGC<br>TGCATTAATGAATCGGCCAACGCGCGGGGAGAGGCGGTTTGCGTATTGGGCGCTCTTCCGCTTCCTCGCTCACTGA<br>CTCGCTGCGCTCGGTCGTTCGGCTGCGGCGAGCGGTATCAGCTCACTCAAAGGCGGTAATACGGTTATCCACAGA<br>ATCAGGGGATAACGCACGAAAGAACATGTGAGCAAAAGGCCAGCAAAAGGCCAGGAACCGTAAAAAGGCCGC<br>GTTGCTGGCGTTTTTCCATAGGCTCCGCCCCCCTGACGAGCATCACAAAAATCGACGCTCAAGTCAGAAG
NS	890	AGCAAAAGCAGGGTGACAAAAACATAATGGATTCCAACACTGTGTCAAGCTTTCAGGTAGACTGCTTCCTTTGGC<br>ATGTCCGCAAACGATTTGCAGACCAAGAACTGGGTGATGCCCCATTCCTTGACCGGCTTCGCCGAGATCAGAAGT<br>CCCTAAGGGGAAGAGGCAGCACTCTTGGTCTGGACATCGAAACAGCTACTCGTGCGGGAAAGCAGATAGTGGAG<br>CGGATTCTGGAGGAGGAATCCGATGAGGCACTCAAAATGACCATTGCTTCTGTGCCGGCTTCACGCTACCTAACT<br>GACATGACTCTTGAAGAAATGTCAAGAGACTGGTTCATGCTCATGCCCAAGCAAAAAGTGGCAGGTTCCCTTTGC<br>ATCAAAATGGACCAGGCAATAATGGACAAGACCATCACATTGAAAGCAAATTTCAGTGTGATTTTTGACCGGCTG<br>GAGACTTTAATACTACTTAGGGCTTTCACAGAAGAAGGAGCAATTGTGGGAGAAATCTCACCATTACCTTCTCTTC<br>CAGGACATACTGATGAGGATGTCAAAAATGCAATTGGGATCCTCATCGGAGGACTTGAATGGAATGATAACACA<br>GTTCGAATCTCTGAAACTCTACAGAGATTCGCTTGGAGAAGCAGTAATGAGGATGGGAGACCTCCACTCCCTTCA<br>AAGCAGAAACGGAAAATGGCGAGAACAATTGAGTCAGAAGTTTGAAGAAATAAGATGGCTGATTGAAGAAGTG<br>CGGCATAAGTTGAAAGTTACGGAGAACAGCTTCGAACAGATAACGTTTATGCAAGCCTTACAACTATTGCTTGAA<br>GTGGAGCAAGAGATAAGAACTTTCTCGTTTCAGTTTATTTAATGATAAAAAACACCCTTGTTTCTACT

Table S3. Estimated evolutionary rates of each gene segments of JX 5-1.

**Genes**	**Substitution rate and 95% HPD (substitution/site/year)**		
	**Mean**	**Lower**	**Upper**
PB2	3.191E-3	2.8197E-3	3.5367E-3
PB1	2.972E-3	2.5965E-3	3.3556E-3
PA	3.3447E-3	2.9327E-3	3.7636E-3
HA	1.1155E-3	9.7201E-4	1.2814E-3
NP	3.9949E-3	3.4007E-3	4.5856E-3
NA	1.8831E-3	1.6086E-3	2.1662E-3
M	2.4272E-3	1.9431E-3	2.8773E-3
NS	1.5112E-3	1.2077E-3	1.8259E-3

Table S4. Virus titer for animal experiments.

Virus	Characteristics	
	EID_{50}	$TCID_{50}$
H3N8	$10^{8.5}$	$10^{2.25}$

Table S5. Virus titers in lung and brain of the infected mice.

$log_{10}(TCID_{50}/mL)$	Lung	Brain
3dpi	2.40 ± 0.34	1.16 ± 0.62
5dpi	2.95 ± 0.57	1.32 ± 0.37
14dpi	-	-

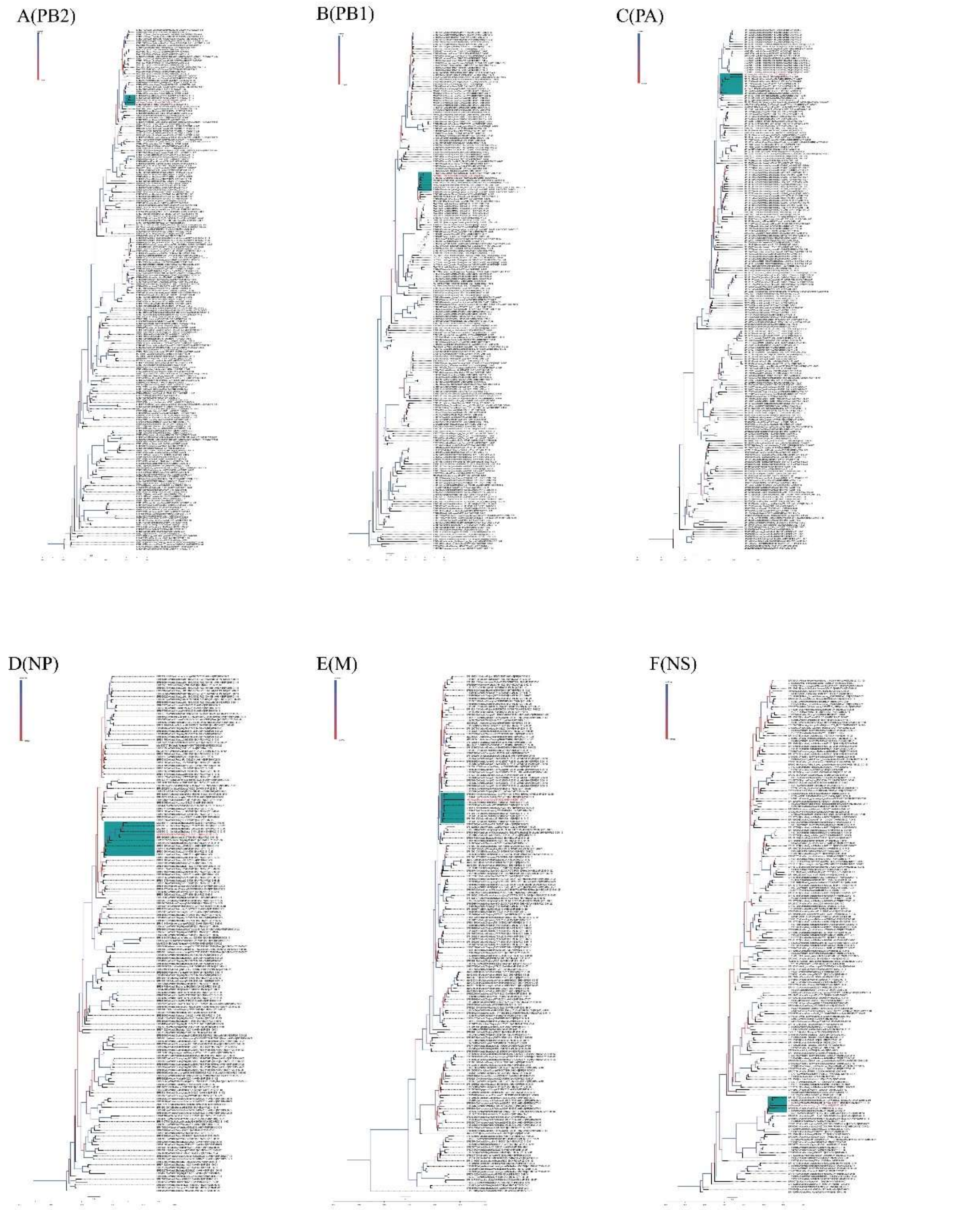

Figure S1. Phylogenetic analysis of PB2 (A), PB1 (B), PA(C), NP(D), M(E), NS(F) of JX 5-1. The phylogenetic trees were constructed using gene sequences identified in NCBI or GISAID Blast analyses. JX 5-1 is marked in red, the closest related sequence is marked in blue. The trees were built using BEAST (v1.8.4) and illustrated using FigTree (v1.4.2).

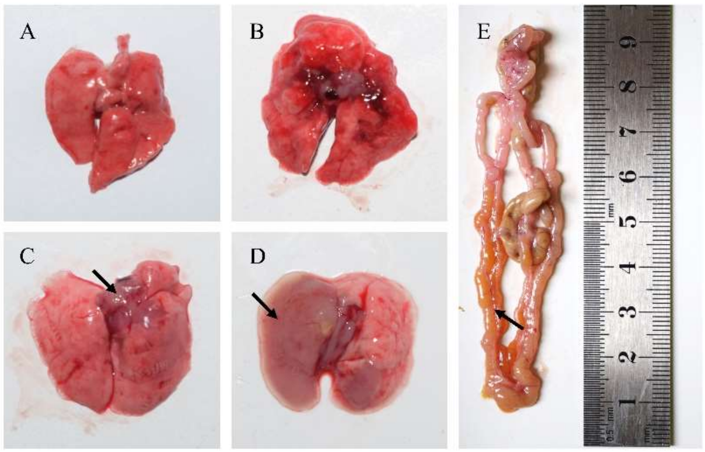

Figure S2. Pathogenicity of lungs and intestine in mice after post-infection. Pathogenicity of lungs: A(Normal), B(3dpi), C(5dpi), D(14dpi). Pathogenicity of intestine: E(14dpi). As indicated by the black arrows in the figure, the lung tissue showed substantial lesions at 5 dpi and 14 dpi, and the intestine had edema at 14 dpi.

Reference

1. E. Hoffmann, J.S., Y. Guan, R. G. Webster and D. R. Perez. Universal primer set for the full-length amplification of all influenza A viruses. Archives of Virology. 2001,*146*, 2275–2289.

2. Kreibich, A.; Stech, J.; Mettenleiter, T.C. ; Stech, O. Simultaneous one-tube full-length amplification of the NA, NP, M, and NS genes of influenza A viruses for reverse genetics. J. Virol. Methods. 2009,*159* (2), 308-310.

3. Dong, C.Y.; Sun, X.N.; Guan, Z.H.; Zhang, M.L. ; Duan, M. Modulation of influenza A virus replication by microRNA-9 through targeting MCPIP1. J. Med. Virol. 2017,*89* (1), 41-48.
